# Editorial: Globally or Regionally Spread of Epidemic Plasmids Carrying Clinically Important Resistance Genes: Epidemiology, Molecular Mechanism, and Drivers

**DOI:** 10.3389/fmicb.2021.822802

**Published:** 2021-12-24

**Authors:** Yi-Yun Liu, Sheng Chen, Vincent Burrus, Jian-Hua Liu

**Affiliations:** ^1^College of Veterinary Medicine, South China Agricultural University, Guangzhou, China; ^2^Guangdong Laboratory for Lingnan Modern Agriculture, Guangzhou, China; ^3^Department of Infectious Diseases and Public Health, City University of Hong Kong, Kowloon, Hong Kong SAR, China; ^4^Département de Biologie, Université De Sherbrooke, Sherbrooke, QC, Canada

**Keywords:** plasmid, resistance gene, epidemiology, transfer mechanism, horizontal spread

Plasmids play a key role in the evolution of bacterial antibiotic resistance by acting as vehicles for horizontal gene transfer. Crucially, some clinically relevant resistance genes, like *bla*_CTX−M_, *mcr-1*, and *bla*_NDM_, are commonly carried by epidemic plasmids (e.g., IncI2, IncX4, IncX3). These resistance plasmids have been disseminated globally to bacterial strains of diverse sources (e.g., animal, human being, foods, and environment). In addition, some epidemic plasmids, such as IncI1 plasmids harboring the *bla*_CTX−M−1_ gene, and the IncF33 plasmids carrying the *bla*_CTX−M−55/65_, *fosA3*, and/or *bla*_KPC−2_ genes, are known to have spread regionally among some European countries and China, respectively. The emergence of bacteria carrying resistance plasmids in various environmental niches is evidence of rapid propagation of antibiotic resistance among bacterial populations of diverse genetic backgrounds. Nevertheless, our knowledge regarding how and to what extent epidemic plasmids carrying clinically relevant resistance genes propels the evolution of antibiotic resistance remains inadequate. Within this topic, 11 articles published recently complement our knowledge of epidemiology, molecular mechanism, and drivers for the dissemination of the epidemic plasmids carrying clinically relevant resistance genes among bacteria from various environmental sources and geographical regions.

The sources of plasmids carrying antibiotic resistance genes as reported in these 11 articles were diverse, comprising human (Fan et al.; Prussing et al.; Wang et al.; Hirabayashi et al.; Huang et al.; Toledano-Tableros et al.), animal (Dor et al.) and food samples (Kurittu et al.). Resistance genes investigated in these works include not only the *bla* variants that encode β-lactamases, which degrade β-lactam antibiotics (Prussing et al.; Wang et al.; Hirabayashi et al.; Huang et al.; Toledano-Tableros et al.), and the *mcr-1* gene that confers resistance to polymyxins (Fan et al.), but also the *lsa*(E) gene that mediates resistance to pleuromutilin, lincosamide, and streptogramin A (PLSA phenotype) (Yan et al.). The studies described in these publications reveal that resistance plasmids of different origins are often structurally related to each other.

Strains of Enterobacteriaceae producing extended-spectrum beta-lactamase (ESBL), plasmid-mediated AmpC-type cephalosporinase (pAmpC) and carbapenemase pose a serious threat to human health. The potential zoonotic origin of ESBL/AmpC-producing bacteria was investigated by two studies (Dor et al.; Kurittu et al.). Kurittu et al. observed ESBL/pAmpC carriage in *Escherichia coli* and/or *Klebsiella pneumoniae* in 14 (7%) of 200 food products collected from 35 countries, as well as 36 (90%) of 40 broiler meat samples. The finding that the dissemination of ESBL/pAmpC genes is mediated by highly transmissible epidemic plasmids (e.g., IncFII and IncI1) highlights the risks of spreading resistant organisms through international trade (Kurittu et al.). An additional article by Dor et al. reported the emergence of ESBL-producing *Salmonella enterica* (ESBL-S) in hospitalized horses in Israel and attributed the surge of CTX-M-3 ESBL-encoding strains mainly to the dissemination of a broad host range plasmid (IncM2) among animal-borne *Salmonella* strains (Dor et al.).

Five studies reported the presence and genetic features of carbapenemase-producing Enterobacteriaceae strains isolated from patients. Prussing et al. investigated the prevalence of KPC-producing bacteria in two healthcare facilities in the United States and reported identical plasmids carrying the *bla*_KPC−2_ gene in four isolates belonging to three bacterial genera (*Citrobacter freundii, K. pneumoniae*, and *E. coli*) in the United States (Prussing et al.). Huang et al. provide key insights into the diverse genetic structures carrying the *bla*_KPC−2_ gene in clinical carbapenem-resistant hypervirulent *K. pneumoniae* (CR-hvKP) strains in Southern China. In their study, the IncF plasmids with a *bla*_KPC_-bearing non-Tn4401 element and the pLVPK-like virulence plasmid were found to be responsible for the propagation of CR-hvKP, especially those of the ST11 type (Huang et al.). Also, horizontal transfer of the *bla*_NDM−1_ gene through IncF-like plasmids among *K. pneumoniae* strains of different sequence types in a tertiary referral hospital in Mexico was reported (Toledano-Tableros et al.). On the other hand, carbapenemase-resistance genes (e.g., *bla*_NDM−5_, *bla*_OXA−48_, and *bla*_OXA−23_) harbored by clinical isolates were reported by Hirabayashi et al.. Based on on-site genomic and epidemiological analyses of carbapenemase-producing Gram-negative bacteria (CPGNB) using portable laboratory equipment, this work provided the first glimpse into the epidemiology of CPGNB in Cambodia and found that two *E. coli* isolates and one *Acinetobacter baumannii* isolate harbored the carbapenemase genes *bla*_NDM−5_ (carried by IncF), *bla*_OXA−48_ (carried by IncX3), and *bla*_OXA−23_ (located in chromosome), respectively (Hirabayashi et al.). In another study from China, Wang et al. reported the recovery of an extensively drug-resistant (XDR) *E. coli* strain from a patient. This XDR *E. coli* strain was found to carry two plasmids: an IncFIB plasmid carrying the *bla*_NDM−5_ gene and an IncFII plasmid carrying a truncated *bla*_TEM_ gene (Wang et al.). These two plasmids were found to be responsible for the resistance phenotypes expressed by this strain, including resistance to all commonly used β-lactam/BLI combinations (Wang et al.).

Rapid dissemination of the plasmid-borne colistin resistance determinant *mcr-1* threatens clinical usage of polymyxins, one of the last-resort antibiotics, to treat multidrug-resistant infections. Fan et al. investigated the origin and genetic characteristics of the *mcr-1* gene in clinical *Salmonella* strains collected from patients in China between 2009 and 2018 (Fan et al.). They reported that the *mcr-1* gene was detectable in six of the 689 *Salmonella* strains (0.87%) collected during this period and that *mcr-1* might be carried by two types of plasmids (IncHI2, *n* = 4; IncI2, *n* = 1) or located in the chromosome (*n* = 1). Besides, recovery of plasmids harboring the *lsa* genes also raised much concern as dissemination of these genes to *Enterococcus faecium* and *Staphylococcus aureus* may further increase the clinical burden due to drug-resistant bacterial infections. Yan et al. collected 96 *E. faecium* strains from one hospital in Beijing in 2013 and found that 46 (47.92%) and two isolates (2.08%) were positive to *lsa*(E) and *lsa*(A), respectively (Yan et al.). In this work, evaluation of the transferability of the *lsa*(E) gene provided the first evidence that *lsa*(E) can be transferred via plasmid conjugation from *E. faecium* to *S. aureus in vitro*.

To better understand how plasmids evolve, Zhang et al. characterized the diversification and evolution history of plasmids belonging to the incompatibility group C (IncC) (Zhang et al.). These researchers identified 20 IncC-positive strains from 870 Enterobacteriaceae strains isolated from food-producing animals in China. Based on four key structural differences in the IncC backbone, 20 IncC plasmids were classified into type 1 (*n* = 4), type 1/2 hybrid (*n* = 15), and type 2 (*n* = 1), respectively. Further analysis of the accessory resistance modules in these plasmids revealed that the various phenotypes expressed by the host strains carrying such plasmids were driven by loss and gain of various genetic modules within the *bla*_CMY_-bearing region and two antibiotic resistance islands (ARI-A and ARI-B), as well as *via* IS*26* or IS*1294*-mediated genetic rearrangements (Zhang et al.).

Plasmids are generally transferred through either conjugation or natural transformation. The transfer of the resistance genes *via* a genetic vehicle (plasmids) has been widely investigated under laboratory conditions; however, research on bacterial plasmid conjugation in the intestinal microbiota remains scarce. A mini-review by Neil et al. describes the molecular mechanisms underlying plasmid conjugation in the gut microbiota. This article summarized, based on currently available evidence, that known conjugative plasmids were not efficiently transmissible among members of the intestinal microbiota. Moreover, these authors discussed the mating-pair stabilization (MPS) mechanisms and factors that influence bacterial conjugation, such as T4SS compatibility, replication compatibility and MPS specificity. Knowledge of bacterial plasmid conjugation *in situ* could facilitate the use of microbiome editing technologies to control antibiotic resistance (Neil et al.).

In summary, articles presented in this Research Topic demonstrated that plasmids are one of the main driving forces in the evolution of bacterial antibiotic resistance and highlighted the importance of collaboration among experts in different fields to tackle antibiotic resistance-related problems. More effort is needed to decipher the molecular mechanisms contributing to the formation and extensive transmission of epidemic plasmids among bacterial pathogens.

## Author Contributions

All authors listed have made a substantial, direct, and intellectual contribution to the work and approved it for publication.

## Funding

This study was supported by grants from National Natural Science Foundation of China (No. 31625026).

## Conflict of Interest

The authors declare that the research was conducted in the absence of any commercial or financial relationships that could be construed as a potential conflict of interest.

## Publisher's Note

All claims expressed in this article are solely those of the authors and do not necessarily represent those of their affiliated organizations, or those of the publisher, the editors and the reviewers. Any product that may be evaluated in this article, or claim that may be made by its manufacturer, is not guaranteed or endorsed by the publisher.

